# Functional Characterization of the Octenol Receptor Neuron on the Maxillary Palps of the Yellow Fever Mosquito, *Aedes aegypti*

**DOI:** 10.1371/journal.pone.0021785

**Published:** 2011-06-30

**Authors:** Alan J. Grant, Joseph C. Dickens

**Affiliations:** Invasive Insect Biocontrol and Behavior Laboratory, Henry A. Wallace Beltsville Agricultural Research Center, Plant Sciences Institute, Agricultural Research Service, United States Department of Agriculture, Beltsville, Maryland, United States of America; Tokyo Medical and Dental University, Japan

## Abstract

**Background:**

1-Octen-3-ol (octenol) is a common attractant released by vertebrates which in combination with carbon dioxide (CO_2_) attracts hematophagous arthropods including mosquitoes. A receptor neuron contained within basiconic sensilla on the maxillary palps of adult mosquitoes responds selectively to 1-octen-3-ol. Recently, an odorant receptor (AaegOR8) known to occur on the maxillary palps was expressed in a heterologous system and demonstrated to be selectively sensitive to (*R*)-(−)-1-octen-3-ol, one of two enantiomeric forms. Lesser responses were elicited by stimulation with the (*S*)-enantiomer and various structural analogs.

**Methodology/Principal Findings:**

Here we characterize the specificity of the octenol receptor neuron in the yellow fever mosquito, *Aedes aegypti* (L.), in vivo using single cell recordings. The octenol neuron is exquisitely sensitive to (*R*)-(−)-1-octen-3-ol; comparable responses to (*S*)-(+)-1-octen-3-ol were elicited only at stimulus doses over 100× that required for the (*R*)-enantiomer. An intermediate response closer to that elicited by the (*R*)-(−)-enantiomer was elicited by racemic 1-octen-3-ol. Small structural changes in (*R*)-(−)-1-octen-3-ol resulted in large decreases in responses. Increases in spike activity were also elicited in the octenol neuron by 2-undecanone, a known repellent; other repellents (DEET, IR3535 and picaridin) were inactive.

**Conclusions/Significance:**

The results of our electrophysiological studies of the octenol receptor neuron in vivo approximates results of a previous study of the octenol receptor (AaegOR8 with its obligate partner Aaeg\ORco) expressed heterologously in *Xenopus* oocytes. By comparison of our current results with those of the heterologous expression study, we conclude that specificity of the octenol receptor neuron can be explained largely by characteristics of the OR alone without other associated proteins present in vivo. Our findings show that repellents may have specific stimulatory effects on receptor neurons and support the notion of repellents as modulators of mosquito odorant receptor activity.

## Introduction

1-Octen-3-ol (octenol), a common attractant emitted by vertebrates, when presented with carbon dioxide (CO_2_) attracts hematophagous arthropods including many biting flies, such as mosquitoes [1], biting midges [2], tsetse flies [3] and tabanids [4]. For some hematophagous insects including the tsetse fly [3] and biting midges [5], octenol alone is attractive. However, for many mosquitoes octenol serves primarily as a behavioral synergist with CO_2_ [1].

**Figure 1:**
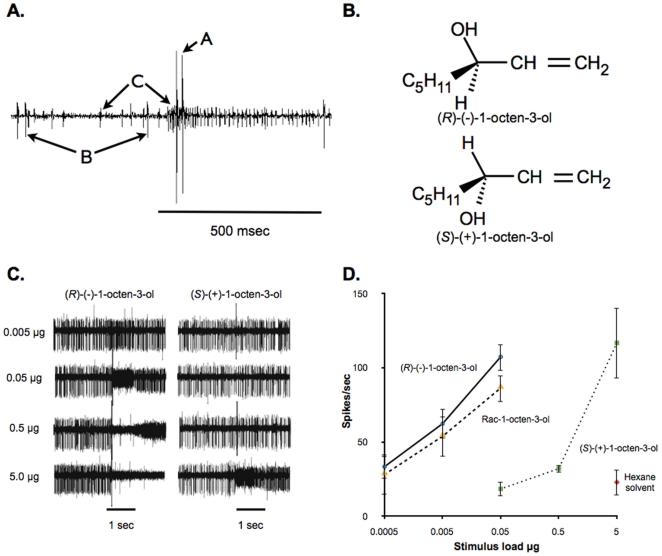
Chiral selectivity of the “C” neuron of maxillary palp basiconic sensilla. (A) Spontaneous activity of neurons associated with a basiconic sensillum on the maxillary palp of a female *Ae. aegypti*. Note the presence of three spike heights: A = largest spike is responsive to CO_2_, B = intermediate spike, and C = smallest amplitude spike is reliably activated by (*R*)-(−)-1-octen-3-ol. Response of “C” neuron is to a low dose of (*R*)-(−)-1-octen-3-ol. (B) Structure of enantiomers of 1-octen-3-ol: (*R*)-(−)-1-octen-3-ol and (*S*)-(+)-1-octen-3-ol. (C) Electrophysiological responses from an individual basiconic sensillum to a 1 sec exposure to increasing stimulus loads of the enantiomers of 1-octen-3-ol. Note at higher loads of (*R*)-(−)-1-octen-3-ol spikes from “C” neuron are not easily discerned from baseline. (D) Dose-response curves constructed from mean responses of “C” neurons to increasing stimulus loads of racemic 1-octen-3-ol (Rac-1-octen-3-ol, n = 4)), and its component enantiomers, (*R*)-(−)-1-octen-3-ol (n = 5), and (*S*)-(+)-1-octen-3-ol (n = 4). Points represented are mean ± SEM.

The detection of octenol is accomplished by receptor neurons housed within sensilla on the maxillary palps. Both sexes of mosquitoes possess basiconic sensilla that contain three neurons; in *Aedes aegypti* these sensilla number about 35 in females and 21 in males [6]. The neuron producing the smallest amplitude spike (“C” neuron) in *Ae. aegypti* responds selectively to octenol [7] (Fig. 1A). The intermediate amplitude spike (“B” neuron) has been reported in *Anopheles gambiae* to respond to octenol [8]. The neuron with the smallest spike amplitude spike (the “C” neuron) in *An. gambiae* responds to various compounds including 2,4,5-trimethyl thiazole [8]. The neuron with the largest spike amplitude (the “A” neuron) responds to CO_2_ [9], [7], [10], [11]. Octenol is a chiral compound and therefore exists in two enantiomeric forms, (*R*)-(−)-1-octen-3-ol and (*S*)-(+)-1-octen-3-ol (Fig. 1B). In field tests, traps baited with (*R*)-(−)-1-octen-3-ol captured more mosquitoes than traps containing (*S*)-(+)-1-octen-3-ol [12]. In single cell recordings in *Culex quinquefaciatus*, a neuron housed in sensilla on the maxillary palps responded preferably to (*R*)-(−)-1-octen-3-ol when compared with (*S*)-(+)-1-octen-3-ol and the racemic mixture [13]. Behavioral preference for a specific enantiomeric form in insects has been known for some time at both the behavioral [14] and physiological levels [15].

The process of odor detection is complex and involves a series of transductory events including the binding of an odorant with a receptor protein (odorant receptor = OR) embedded in the lipid bilayer of a sensory neuron. With the advent of molecular methods, recent work has begun to further elucidate these events, especially with regards to specificity of ORs for biologically-relevant ligands [16], [17], [18], [13], [19], [20]. Using a heterologous expression system, Bohbot and Dickens [21] recently demonstrated the specificity of the odorant receptor AaegOR8 (expressed with its obligate partner Aaeg\Orco) for (*R*)-(−)-1-octen-3-ol. More recently, 2-undecanone, a known repellent, was also shown to activate this OR [22], [23].

In our present study, we continue these investigations by recording *in vivo* responses from peripheral sensory neurons contained in basiconic sensilla on the maxillary palps of female *Ae. aegypti* to graded doses of the octenol enantiomers as well as structurally similar compounds and behavioral repellents. We compare these *in vivo* recordings from the peripheral sensory system with *ex vivo* studies of the ORs previously reported [21], [22], [23].

## Results

### The “C” neuron is preferentially stimulated by (*R*)-(−)-1-octen-3-ol

In general, electrophysiological recordings of neural activity elicited by increasing stimulus loads of odorants revealed increased activity of the “C” neuron (the smallest amplitude spike)(Fig. 1A and 1C). Dose-response curves for single unit responses to (*R*)-(−)-1-octen-3-ol and (*S*)-(+)-1-octen-3-ol were similar in shape, but the curve for the (*S*)-(+)-enantiomer was shifted to the right by approximately three log steps (Fig. 1D). The dose response curve for racemic 1-octen-3-ol paralleled the curve for the (*R*)-(−)-enantiomer only shifted slightly to the right. If we assume that the enantiomeric mixture of the racemic sample is made up of approximately 50% (*R*) and 50% (*S*), then the total amount of the (*R*) enantiomer in the racemic mixture is half that of the pure (*R*) sample. Therefore, as expected, the racemic response curve in Fig. 1D is shifted by approximately ½ a log unit. Saturation levels were not clearly reached for either racemic 1-octen-3-ol or the enantiomers and other compounds because at high firing rates spikes from this neuron were driven to levels not discernible from the baseline, e.g. (*R*)-(−)-1-octen-3-ol at 0.5 µg dose (Fig. 1C).

Since we do not know the actual odorant concentration at the levels tested, the data are expressed as doses loaded onto filter paper strips placed in the stimulus cartridge vs. spikes/second. Although the purity of these samples was high, the possibility exists that some of the response to (*S*)-(+)-1-octen-3-ol could be due to a very small amount of the (*R*)-enantiomer. As was observed with the ORs expressed *ex vivo* [21], the chirality at the third position of the molecule is clearly important for responsiveness.

Temporal discharge patterns of responses to the (*R*) and (*S*) enantiomers of 1-octen-3-ol during the 1 sec stimulus period did not clearly differ except that the threshold responses to the (*S*)-enantiomer were offset by nearly 3 log steps (data not shown).

### Chain length, unsaturation, location of chiral center, and functional group are important for activity

**Figure 2:**
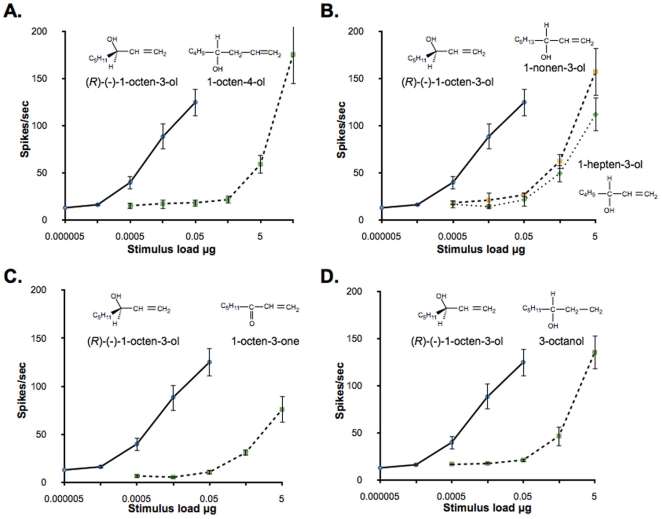
(*R*)-(−)-1-octen-3-ol strongly preferred over related analogs. Dose-response relationships from the “C” neuron in maxillary palp sensilla of female *Ae. aegypti* in response to increasing doses of (*R*)-(−)-1-octen-3-ol (n = 5) and structurally similar chemicals. (A) Movement of chiral center from “3” to “4” position, 1-octen-4-ol (n = 4). (B) Changes in carbon chain length: 1-hepten-3-ol (n = 4) and 1-nonen-3-ol (n = 3). (C) Change in functional group with loss of chiral center, 1-octen-3-one (n = 4). (D) Saturation of double bond, 3-octanol (n = 4). Points represented are mean ± SEM.

For comparative purposes, we used the same set of odorants to test the sensitivity of the *in vivo* receptor neurons with the previously reported sensitivity of the AaegOR8 expressed heterologously [21]. In the neural recordings, while the structural analogues elicited responses, substantially higher doses were required for comparable responses to stimulation with the (*R*)-(−)-1-octen-3-ol (Fig 2. A–D). Moving the chiral center to C4 (1-octen-4-ol) reduced the effectiveness of the stimulus by approximately 3 log steps (Fig. 2A). Shortening (1-hepten-3-ol) or lengthening (1-nonen-3-ol) the carbon chain also greatly reduced the effectiveness of the stimulus as compared with the (*R*)-(−)-1-octen-3-ol (Fig. 2B). The achiral 1-octen-3-one was approximately 3 orders of magnitude less effective in stimulating the octenol neuron as compared with the (*R*)-enantiomer of octenol (Fig. 2C). The importance of the double bond was demonstrated by the shifted dose response curve obtained for 3-octanol (Fig. 2D).

### 2-Undecanone, a known repellent, activates the “C” neuron

**Figure 3:**
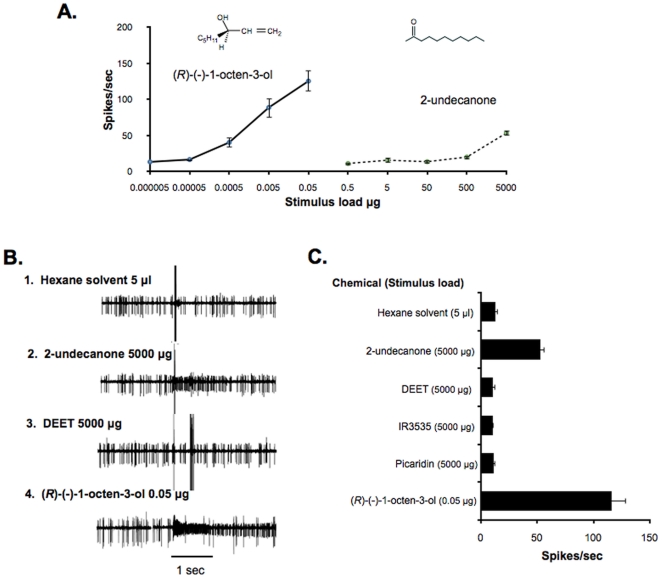
2-Undecanone, a known repellent, activates the “C” neuron. (A) Dose-response relationship from the “C” neuron in maxillary palp sensilla of female *Ae. aegypti* in response to increasing doses of (*R*)-(−)-1-octen-3-ol (n = 5) and 2-undecanone (n = 6). (B) Electrophysiological responses from an individual basiconic sensillum on the maxillary palp of a female *Ae. aegypti* to a 1 sec exposure to: 1. hexane solvent, 2. 2-undecanone 5000 µg, 3. DEET 5000 µg, and 4. (*R*)-(−)-1-octen-3-ol 0.05 µg. (C) Mean number of spikes (±SEM; n = 5) from “C” neurons of female *Ae. aegypti* basiconic sensilla to stimulation with 5000 µg doses of hexanes, 2-undecanone, DEET, IR3535, picaridin and to 0.05 µg dose of (*R*)-(−)-1-octen-3-ol. Points represented are mean ± SEM. “A”, “B”, and “C” with arrows designate three spike classes present in recordings from the basiconic sensilla.

Thresholds for dose response curves for (*R*)-(−)-1-octen-3-ol (0.0005 µg) and the known insect repellent 2-undecanone (500 µg) were separated by nearly 7 log steps (Fig. 3A). However, the octenol sensitive neuron was reliably activated by 2-undecanone at 5000 µg source load (Fig. 3B and 3C) while other insect repellents, e.g., DEET, IR3535 and picaridin were inactive at this dose.

## Discussion

Results of the *in vivo* study of the octenol receptor neuron on the palps of female *Ae. aegypti* presented here are similar to those obtained in an *ex vivo* study in which the odorant receptor for octenol AaegOR8 was expressed heterologously in *Xenopus* oocytes [21]. In both cases, the sensitivity of the systems was offset to similar degrees, even though the mode of stimulus presentation was different, i.e., the compounds dissolved in aqueous solution as compared to the volatile released into the air from filter paper. However, dose response curves for the various analogs were offset by two log steps in the heterologous expression system, while those obtained for the same compounds in our current *in vivo* study were offset by approximately three log steps. This increased difference in sensitivity for the cognate agonist (*R*)-(−)-1-octen-3-ol relative to that for closely related compounds might be attributed to other elements present in the *in vivo* system such as odorant binding proteins [24], [25].

Although one clearly would expect ORs to influence olfactory receptor neuron output, similarities between the *in vivo* results presented here and *ex vivo* studies reported previously [21] suggest that ORs play the major role in determining response. These data imply that the events occurring at the protein membrane level are sufficient to explain the neurophysiological responses as measured in spike output by the sensory neurons.

Decreased responses to structural analogues of (*R*)-(−)-1-octen-3-ol demonstrate the importance of certain chemical features in the sensitivity of neuronal responsiveness. Features such the chain length, position of unsaturation, functional groups and enantiomeric composition are important in eliciting responses from the peripheral system.

The mode of action of repellants is currently a topic of much research [13], [22], [23], [26]. Recently, we showed that DEET and other repellents can have multiple effects on specific ORs expressed heterologously in *Xenopus* oocytes [22], [23]: *agonistic or stimulatory effects* when presented alone to the OR and *antagonistic or inhibitory effects* when presented in the presence of the OR's cognate agonist. In those studies, 2-undecanone activated AaegOR8, while DEET, IR3535 and picaridin did not activate this OR, but rather activated another OR, AaegOR2. Data presented here support those studies as 2-undecanone elicited spike activity from the octenol receptor neuron while the other repellents did not elicit spikes (Fig. 3A and 3B). The fact that 2-undecanone only stimulated the octenol neuron at doses much higher doses than its cognate agonist (*R*)-(−)-1-octen-3-ol is consistent with the high concentrations needed for activity of insect repellents [27].

In conclusion, we have shown that the octenol receptor neuron in the basiconic sensilla on the maxillary palps of *Ae. aegypti* is exquisitely sensitive to (*R*)-(−)-1-octen-3-ol; responses of this neuron to structural analogs of this cognate stimulus are greatly reduced. 2-Undecanone, a known repellent, stimulates the octenol receptor at a high dose, while other repellents are inactive. These results are consistent with prior two-electrode voltage clamp studies of the octenol receptor AaegOR8 expressed heterologously in *Xenopus* oocytes [21], and suggest an important role of ORs in determining the sensitivity and specificity of the octenol receptor neuron and its modulation by a known repellent.

## Materials and Methods

### Insects

All electrophysiological recordings were conducted on female *Ae. aegypti* (Liverpool strain). Mosquitoes were housed in an insectary maintained under 12∶12 L∶D at approximately 20°C. Egg sheets were obtained on a regular basis from either The Walter Reed Army Institute of Research in Silver Springs, MD or The Center for Medical and Veterinary Entomology, USDA, ARS in Gainesville, FL, USA. Larvae were fed ground fish food (Tetramin®). Insects were segregated following pupation and adult emergence dates were recorded. Adult mosquitoes were fed *ad lib* 10% sucrose in water.

### Electrophysiological Recordings

Single unit electrophysiological responses were recorded from basiconic sensilla on the maxillary palps of 5–10 day-old females using standard methods [9], [10], [28]. Mosquitoes were secured to a glass plate and maxillary palps were positioned to allow optimal electrode access to sensilla. Electrodes made of 125 µm tungsten wire (Small Parts Co., Lexington, KY, USA) were electrolytically sharpened to tip diameters of less than 1 µm. The indifferent electrode was placed in the compound eye; the recording electrode was positioned at the base of an individual sensillum. Following insertion of the recording electrode, the preparation was allowed to “stabilize” for 15 min. If at that point the signal-to-noise ratio was adequate for reliable separation of action potential classes, the preparation was exposed to 1 sec pulses of odorants. Signals were amplified and filtered (bandpass 300 hz to 1000 hz) with a Grass P15D amplifier (Grass Instrument Corp., Quincy, MA USA). Data were collected, stored and analyzed with AutoSpike software (Syntech, Kirchzarten, Germany) using a microcomputer.

### Odorant Stimulation

Odorants were serially diluted in spectrophotometric-grade hexanes; 5 µl aliquots of the desired odorant concentration were pipetted onto a filter paper strip held in a modified glass capillary tube. Stimulus cartridges were used only once. The carrier gas was compressed air (Ultra Zero Grade; >0.5 ppm CO_2_; 665 mls/min). Two parallel air lines were directed over the preparation: odorant delivery was accomplished by switching between a purge stream and a stimulus-laden stream using a modified Syntech CS-55 Stimulus Controller (Syntech, Kirchzarten, Germany). Stimulations were separated by approximately 3 min during which the preparation was bathed in Ultra Zero grade compressed air.

### Experimental Odorants

(*R*)-(−)-1-Octen-3-ol [99.6% (*R*)] and (*S*)-(+)-1-octen-3-ol [99.9% (*S*)] were obtained from Bedoukian Research, Inc., Danbury, CT, USA. Racemic 1-octen-3-ol (>98%) was obtained from Fluka Chemical Corp., Milwaukee, WI, USA. 1-Octen-4-ol (99%), 1-nonen-3-ol (98%) and 1-octen-3-one (97%) were obtained from Alfa Aesar, Ward Hill, MA, USA. 1-Hepten-3-ol (97%) and 3-octenol (99%) were obtained from Sigma-Aldrich, St Louis, MO, USA. The repellents used in this study, their source and purity were: DEET (N,N-diethyl-3-methylbenzamide, 99.2%), Aldrich, Milwaukee, WI USA; picaridin [2-(2-hydroxyethyl)-1-piperidine carboxylic acid 1-methylpropyl ester, >98%], LANXESS, Pittsburgh, PA, USA; IR3535 (3-[N-Butyl-N-acetyl]-aminopropionic acid ethyl ester, >95%), Merck, Rahway, NJ, USA; and 2-undecanone (99%), Aldrich, Milwaukee, WI, USA.
